# Slow and Steady: A Slowly Progressing Neuroendocrine Tumor

**DOI:** 10.14309/crj.0000000000001147

**Published:** 2023-09-21

**Authors:** Christie Zheng, Reem Al Shabeeb, Dipam Shah, Nitin Sardana

**Affiliations:** 1 University of Virginia School of Medicine, Charlottesville, VA; 2 Department of Internal Medicine, Inova Fairfax Medical Campus, Falls Church, VA; 3 Gastro Health, Virginia Division, Fairfax, VA

**Keywords:** neuroendocrine tumor, metastatic NET

## Abstract

Neuroendocrine tumors (NETs) are rare malignant tumors that arise from neuroendocrine cells throughout the body, most commonly in the gastrointestinal and respiratory tracts. We report a case of well-differentiated grade 2 NET with a computed tomography scan showing multiple liver lesions consistent with the liver lesions seen 11 years before diagnosis. This case highlights the possibility of an indolent or prolonged clinical course of metastatic NET with an unknown primary vs primary hepatic NET.

## INTRODUCTION

Neuroendocrine tumors (NETs) are malignant tumors that arise from neuroendocrine cells throughout the body, most commonly in the gastrointestinal (GI) and respiratory tracts.^1^ Within the GI tract, NETs are most commonly located in the small intestine (44.7%), rectum (19.6%), appendix (16.7%), and colon (10.6%).^2^ NETs are rare tumors with an annual incidence of 6.98 per 100,000 persons, which has been steadily increasing in recent years.^3,4^ NETs are histologically characterized by the degree of differentiation based on features such as tumor cell organization and nuclei morphology.^1^ In addition, NETs can be further categorized into grade 1–3 by the Ki-67 index, with grade 3 NETs having the highest mitotic activities.^1^

NETs can be asymptomatic initially because the liver can inactivate hormones secreted by the tumors. Therefore, symptoms may not occur until the tumors have metastasized to the liver. As a result, many patients present with advanced disease at the time of diagnosis.^5^ Patients often undergo multiple visits to primary care physicians or specialists before receiving a diagnosis, and the time from symptom onset to diagnosis can be as long as 9.2 years.^6–8^ Although NETs can have a more indolent course compared with other epithelial carcinomas, metastatic NETs have been shown to have significantly worse prognosis compared with localized diseases.^3,4^

## CASE REPORT

**Figure 1.**
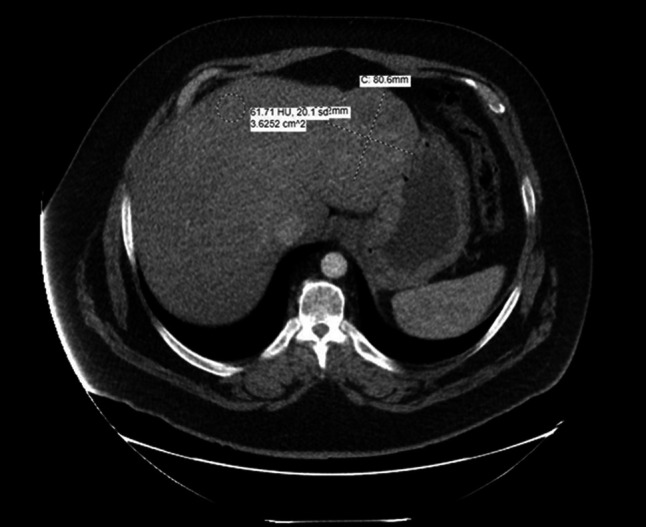
Patient's first computed tomography scan, 7 years prior.

A man in his mid-40s with a family history of colon cancer presented with intermittent rectal bleeding. Records revealed that 7 years before this visit, the patient had an abdominal and pelvic computed tomography (CT) scan because of abdominal pain. CT at that time showed multiple indeterminate liver lesions, which the radiologist suspected to represent focal nodular hyperplasia (FNH), although the enhancement pattern was not characteristic (Figure 1). The patient was lost to follow-up after that scan. Workup of the patient's current symptom of hematochezia included a colonoscopy, although it was limited to the ascending colon because of tortuosity in the colon. The colonoscopy was only notable for internal hemorrhoids. Repeat colonoscopy was attempted 6 months later with hopes of reaching the cecum, though this was also unsuccessful. During this time, the patient was started on phentermine for weight management and had a 50 pound weight loss. He also started experiencing intermittent nonbloody loose stool occurring 2–3 times a day on average and occasional nocturnal diarrhea, abdominal pain, nausea, decreased appetite, and fatigue. Symptoms persisted despite discontinuation of phentermine.

To complete the luminal workup, cross-sectional imaging was obtained with CT colonography. While this did not show any colonic lesions, it did show a liver with nodular contour and several hypodense liver lesions, with the largest lesion measuring up to 12.4 × 11.2 cm. In addition, there was wall thickening of the proximal aspect of the lesser curvature of the stomach and a 1.7 cm sclerotic lesion at the right posterior seventh rib. The patient denied any known personal or family history of liver disease, but did note a family history of colon cancer in his paternal grandfather and breast cancer in his mother and maternal cousin. He did not have any significant history of alcohol use.

**Figure 2.**
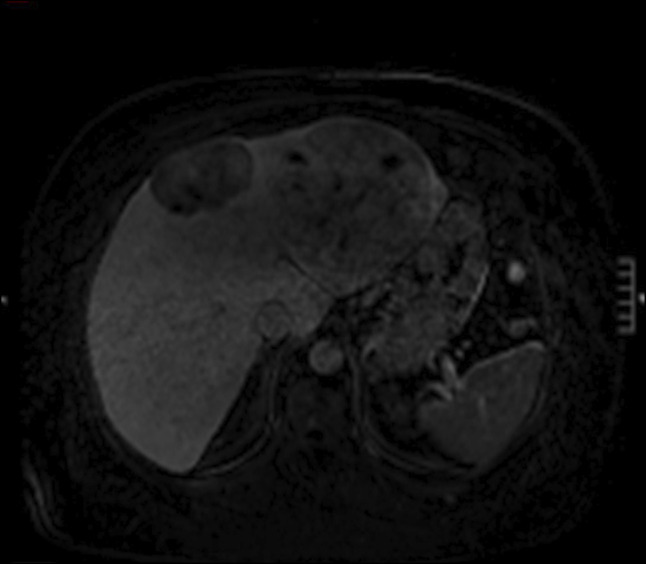
Magnetic resonance imaging shows multiple heterogeneously enhancing hepatic masses.

Owing to the nodular appearance of the liver, a chronic liver disease laboratory panel was obtained. Liver function tests were normal. Viral hepatitis panel was only positive for hepatitis A antibody. The remainder of the chronic liver disease workup was also unrevealing. The patient underwent magnetic resonance imaging with and without contrast, which showed multiple heterogeneously enhancing hepatic masses (Figure 2). The largest lesion was in the left hepatic lobe at 13.1 × 11.4 cm, which was increased from around 8 cm compared with the initial CT scan in 2011.

An ultrasound-guided liver biopsy of the right hepatic lobe lesion was performed. Pathology of the biopsy showed low-grade NET with a KI-67 proliferation rate of 10%. Immunohistochemical phenotype was positive for caudal-type homeobox 2, synapsin, and chromogranin A. The patient then underwent gallium positron emission tomography, which showed multifocal somatostatin-avid liver masses with the bulk of the disease contained in the left lobe and 2 smaller foci in the right lobe. There was no evidence of primary or other metastatic lesions. Esophagogastroduodenoscopy was also performed because of wall thickening of the stomach on CT colonography. Biopsies from the esophagogastroduodenoscopy showed gastric hyperplastic polyps and fundic gland polyps, but were negative for dysplasia or NETs. The patient was treated with pantoprazole with improvement of his GI symptoms and fatigue. The bleeding was self-limited and was suspected to be due to benign anorectal disease. Repeat colonoscopy is to be considered if bleeding persists despite conservative management.

Based on imaging and pathology results, the patient was diagnosed with stage IV pM1 well-differentiated neuroendocrine carcinoma. Given lack of clear source, the differential included a primary hepatic NET vs an unknown primary with liver metastases. He was followed by gastroenterology, medical oncology, and surgery. Preoperative tumor markers showed normal plasma 5-hydroxyindoleacetic acid at 20 ng/mL, elevated pancreastatin at 565 ng/mL, elevated chromogranin A at 1,873 ng/mL, elevated pancreatic polypeptide at 439 pg/mL, and elevated serum serotonin at 309 ng/mL.

Owing to the patient's family history of multiple cancers, he was also evaluated by genetics. A 77-gene CancerNext-Expanded panel was negative.

Around 2 months after the pathology-confirmed diagnosis, the patient underwent elective surgical debulking of the tumors with left lateral hepatectomy of segments 4 and 5 and liver ablation of segment 8. More than 95% of the tumor bulk was successfully removed. Two deeper lesions on the right side were not resected because of their close proximity to the right portal pedicle. Surgical pathology again demonstrated grade 2 well-differentiated neuroendocrine neoplasm.

Two months after the surgery, magnetic resonance imaging showed no new lesions and the inferior right hepatic lobe lesions had decreased in size. Tumor markers were downtrending, with chromogranin A at 165 ng/mL and pancreatic polypeptide at 283 pg/mL. At the 3-month postsurgical follow-up, the patient is alive and doing well.

## DISCUSSION

NETs encompass a heterogeneous group of tumors with varying histological characteristics and, often, nonspecific clinical presentations. Although NETs are rare, the incidence rate has seen a 6.4-fold increase from 1973 to 2012, which has been attributed to the increased detection of early-stage diseases and the use of screening endoscopic procedures.^3,4,9^ In this article, we report a case of well-differentiated neuroendocrine neoplasm in the liver. The differential included a primary hepatic NET and a NET with unknown primary with liver metastases that were present on CT imaging 11 years before the diagnosis and surgical resection. Given the similarities in their presentation, primary hepatic NET and NET with unknown primary are both possibilities and difficult to determine which the patient had definitively. The initial impression of the liver lesions was possibly FNH, although the enhancement was atypical. When the patient presented to the clinic and further imaging redemonstrated the lesions, it was initially believed to be reassuring that the lesions were present years ago. The diagnosis of NET was eventually made after a liver biopsy. Given that there are multiple lesions, there exists a possibility that the patient has FNH in addition to the neuroendocrine lesions. This patient's presentation, along with the increased incidence of NETs, highlights the need to consider NETs as a part of the differential diagnoses for liver lesions, even for the ones that are present for a long time.

Delays in diagnosis are not uncommon for NETs. Reported duration from initial symptom onset to diagnosis ranges from 54 months to 9.2 years.^6,7,10^ Many factors contribute to the diagnostic delay, including the rarity of the disease and the nonspecific symptoms that can mimic other conditions, with irritable bowel disease being a common misdiagnosis.^7^ Pain, GI symptoms, and fatigue are the most frequently reported initial symptoms experienced by patients.^6,11^ In addition, many patients can be asymptomatic with incidental diagnosis from screening colonoscopy or imaging performed for other purposes.^11^ The patient in our case experienced several of the aforementioned nonspecific symptoms, including abdominal pain, diarrhea, nausea, decreased appetite, and fatigue. It was difficult to discern whether his weight loss was associated with the tumor in the setting of concurrent phentermine use. His GI symptoms could also be attributed to gastritis, which further highlights the challenge of diagnosing NETs based on the nonspecific symptoms. Previously, Basuroy et al suggested greater use of imaging especially for older patients with red flag symptoms, including weight loss.^6^ In addition, the North American Neuroendocrine Tumor Society has published guidelines with detailed descriptions of symptoms that should raise concerns for NETs and the recommended biochemical testing, genetic testing, and imaging for each subtype of NETs.^7^

It is difficult to determine definitively whether this case represents metastatic disease vs primary hepatic NET. The most unique aspect of our case is that the patient had evidence of liver metastases 11 years before surgical resection. There has been a report of a patient with primary hepatic NET who survived for 26 years before surgical resection, but there are no similar published cases on patients with metastatic NETs.^12^ Although NETs are considered to be more indolent than other carcinomas, metastatic diseases, seen in around 21% of patients at diagnosis, are still associated with poorer outcomes.^9^ Yao et al reported a median survival of 33 months in patients diagnosed with low-grade NETs but with distant diseases, including liver metastasis, compared with 223 months in patients with localized disease.^4^ Similarly, Hallet et al reported a 10-year overall survival of 68.2% for patients without metastasis and 17.5% for patients with metastasis at presentation.^9^ In addition, having metastases with unknown primary sites is associated with worse prognosis compared with patients with known primary sites.^5^ The patient in this case survived for 11 years with metastatic NET and unknown primary site, which suggests the possibility of having an indolent course even with distant disease and unknown primary site. The variability in the progression of NETs can increase difficulties for clinicians to consult patients on prognosis. A nomogram was previously created for small intestinal NETs to help with determining prognosis.^13^ More research is needed in this area to provide tools to estimate disease progression based on specific risk factors.

In conclusion, we report a case of grade 2 well-differentiated NET with liver metastases present 11 years before the diagnosis and surgical resection. Given the nonspecific presenting symptoms and variable courses of progression, more research is needed on improving early detection and predicting prognosis.

## DISCLOSURES

Author contributions: All authors provided final approval of the version to be published. C. Zheng led the drafting of the case report and interpretation of the case. R. Al Shabeeb and D. Shah contributed to the conception of the work and provided critical revision. N. Sardana led the conception of the work, interpretation of the case, and critical revision of the case report and is the article guarantor.

Financial disclosure: None to report.

Informed consent was obtained for this case report.
